# The long noncoding RNA TUG1 is required for TGF-β/TWIST1/EMT-mediated metastasis in colorectal cancer cells

**DOI:** 10.1038/s41419-020-2254-1

**Published:** 2020-01-27

**Authors:** Xuning Shen, Xiu Hu, Jiayan Mao, Ying Wu, Hao Liu, Jian Shen, Jiayin Yu, Wei Chen

**Affiliations:** 1 Department of Gastrointestinal Surgery, Affiliated Hospital of Jiaxing College, Jiaxing, Zhejiang, China; 2 Cancer Institute of Integrated Traditional Chinese and Western Medicine, Zhejiang Academy of Traditional Chinese Medicine, Tongde Hospital of Zhejiang Province, Hangzhou, Zhejiang, China; 3 Department of Pathology, Affiliated Hospital of Jiaxing College, Jiaxing, Zhejiang, China

**Keywords:** Cancer, Cancer, Cell biology, Cell biology

## Abstract

Colorectal cancer (CRC) is one of the leading causes of cancer death worldwide, and metastasis is the major cause of CRC-related mortality. Transforming growth factor-beta (TGF-β) has a central role not only in the regulation of the normal colon but also in the development and metastasis of CRC. However, TGF-β is not considered an ideal therapeutic target because it shows both pro-tumorigenic and anti-tumorigenic activity, depending on the tumor stage. Therefore, it is important to find a downstream signaling component of TGF-β that can be targeted to impair CRC metastasis. Here, we show that TGF-β promotes CRC migration and upregulates the expression of long-noncoding RNA Taurine Upregulated Gene 1 (TUG1). TUG1 knockdown inhibited migration, invasion, and epithelial–mesenchymal transition (EMT) of CRC cells in vitro, and reduced CRC lung metastasis in vivo. TGF-β induced metastasis, and TUG1 knockdown inhibited these effects. In addition, TGF-β could not reverse the anti-metastasis effects of TUG1 knockdown. These data demonstrate that TUG1 is a downstream molecular of TGF-β. Moreover, TWIST1 expression was increased with TGF-β treatment, and TUG1 knockdown decreased TWIST1 expression in CRC cells. TWIST1 knockdown inhibited invasion and EMT in CRC cells; these effects were not changed by simultaneous TUG1 knockdown, indicating that TWIST1 is a downstream mediator of TUG1. Moreover, TUG1 was significantly overexpressed in CRC patients. In conclusion, TGF-β promotes metastasis of CRC via a TUG1/TWIST1/EMT signaling pathway. TUG1 may be a promising drug target to inhibit TGF-β pathway activation in the treatment of CRC.

## Introduction

Colorectal cancer (CRC) is the third most commonly diagnosed form of cancer in both of men and women, accounting for ~ 1 in 10 cancer cases and deaths^1,2^. Prognosis in CRC heavily depends on stage at diagnosis, and whereas survival among patients with local or regional disease has been improving, prognosis for patients with metastatic disease remains dismal^3^. About 25% of CRC patients present with metastatic disease at the time of diagnosis, and 20–25% of patients with local-regional disease will develop metastasis, resulting in a high overall mortality rate (40–45%)^4^. Surgery, chemotherapy, and radiation are three main therapeutic approaches for metastatic CRC^5^. Surgery is usually not an option for patients with advanced CRC, and chemotherapy that lacks selectivity for tumor cells is generally the most effective treatment^5–7^. Given this, further understanding of the molecular mechanisms underlying CRC progression are urgently required to develop better treatments and improve disease prognoses.

Epithelial–mesenchymal transition (EMT) has an important role in promoting the invasion and metastasis of CRC cells^8^. During EMT, epithelial cells lose epithelial characteristics and acquire a mesenchymal, highly invasive phenotype^9^. Transforming growth factor β (TGF-β) is a major inducer of EMT, and activates many transcriptional regulators, such as TWIST1, TWIST2, ZEB1, ZEB2 (SIP1), Snail1 (Snail), and Snail2 (Slug), leading to the downregulation of E-cadherin expression^10^. TWIST1 expression was observed in 86.1% of CRC tissues, and at significantly higher levels than Snail or Slug^8,11^. However, TGF-β can also suppress tumor cells by inducing cell-cycle arrest and apoptosis, which hinders the application of anti-TGF-β treatments in cancer^12^. Therefore, it is important to develop novel strategies to regulate the downstream signaling components of TGF-β.

Long noncoding RNAs (lncRNAs) are a class of RNA transcripts, > 200 nucleotides in length, which lack protein-coding capacity^13^. It has been demonstrated that lncRNAs play crucial roles in cancer progression, impacting proliferation, apoptosis, migration, invasion, and metastasis^14^. The lncRNA Taurine Upregulated Gene 1 (TUG1) is upregulated in CRC cells and clinical samples and promotes metastasis by affecting EMT^15^. However, the mechanism of how TUG1 affects EMT remains unknown. Moreover, it is unclear whether TUG1 is involved in TGF-β-induced EMT and metastasis in CRC.

In this study, we demonstrate that TGF-β promotes CRC migration and upregulation of TUG1 expression. Knockdown of TUG1 suppressed CRC cell migration, invasion and EMT in vitro, and reduced CRC lung metastasis in vivo. Furthermore, TGF-β induced metastasis via the TWIST1/EMT signaling pathway and, TUG1 knockdown inhibited these effects. These data demonstrate that TGF-β promotes metastasis of CRC via a TUG1/TWIST1/EMT signaling pathway. These results suggest that targeting TUG1 may be an effective strategy to inhibit CRC metastasis.

## Results

### TUG1 is associated with TGF-β-induced metastasis in CRC cells

**Fig. 1:**
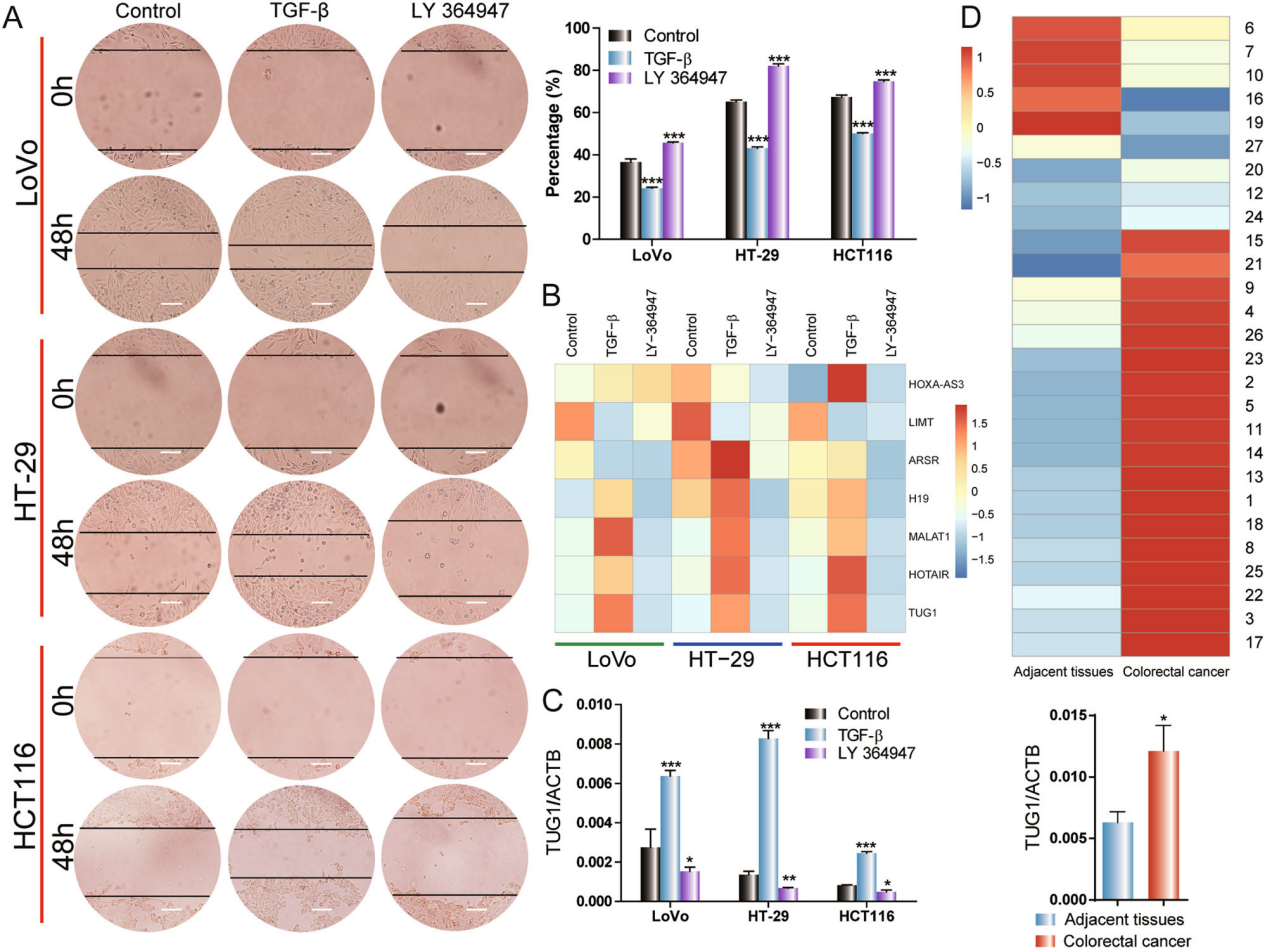
TUG1 expression is associated with active TGF-β signaling and mobility in CRC cells. **a** LoVo, HT-29, and HCT116 cells were treated with 3 ng/ml TGF-β or 2 μm LY 364947. Scale bar, 50 μm. The ratio between the residual gap at 48 h and the initial gap at 0 h was calculated, compared with the control group: ***P < 0.001. **b** The brightness of blue and red in heatmap, respectively, depict the downregulation and upregulation of the genes compared with the controls. The color bar represents a log2 scale. **c** The transcriptional level of TUG1 in LoVo, HT-29, and HCT116 cells was detected by qRT-PCR. *P < 0.05, **P < 0.01, and ***P < 0.001, versus control. **d** The expression of TUG1 was detected in CRC and adjacent tissues by qRT-PCR, *P *<* 0.05.

To identify an lncRNA that is a downstream mediator of TGF-β signaling, LoVo, HT-29, and HCT116 cells were treated with TGF-β or LY 364947 (TGF-β inhibitor). The migration of CRC cells in the TGF-β treatment group was significantly higher than the control group, and LY 364947-treated CRC cells exhibited significantly lower migration (Fig. 1a). In addition, cell viability assay showed that the indicated concentration of TGF-β or LY 364947 did not affect cell proliferation (Fig. S1). Quantitative real-time polymerase chain reaction (qRT-PCR) was used to detect the expression of several lncRNAs. Treatment with TGF-β upregulated TUG1 expression, and LY 364947 treatment downregulated TUG1 expression (Fig. 1b, c). We evaluated TUG1 expression of clinical CRC samples and paired adjacent normal tissue from 27 patients. TUG1 expression was higher in CRC tissues (77.78%, 21/27) than adjacent normal tissue (Fig. 1d). These results demonstrated that TUG1 is downstream of TGF-β signaling, and that overexpression of TUG1 may enhance the migration of CRC cells.

### TUG1 is critical for migration and invasion in CRC cell lines

**Fig. 2:**
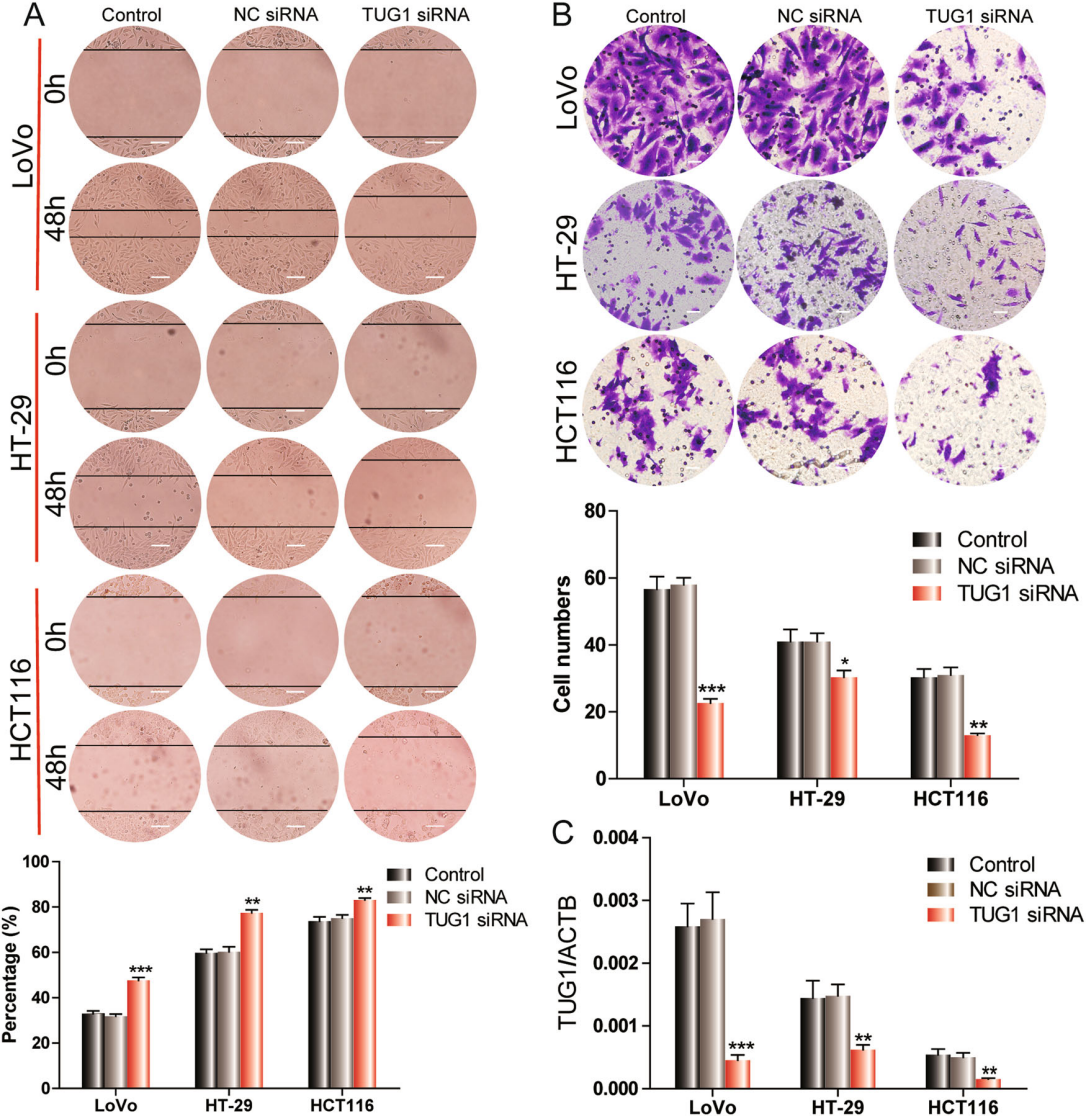
TUG1 promotes migration and invasion in CRC cell lines. **a** Wound-healing assay of LoVo, HT-29, and HCT116 cells transfected with NC-siRNA or TUG1 siRNA, **P < 0.01 and ***P < 0.001, versus NC-siRNA. Scale bar, 50 μm. **b** Transwell invasion assays were used to assess the invasive ability of CRC cells transfected with NC-siRNA, TUG1 siRNA. *P < 0.05, **P < 0.01, and ***P < 0.001, versus NC-siRNA. Scale bar, 100 μm. **c** QRT-PCR was used to confirm the effects of TUG1 knockdown after transfection of TUG1 siRNAs. **P < 0.01 and ***P < 0.001, versus NC-siRNA.

To determine whether TUG1 activation is responsible for CRC metastasis, we used siRNA to knockdown TUG1 expression in CRC cells. The expression of TUG1 siRNA significantly reduced TUG1 expression in CRC cells (Fig. 2c). TUG1 silencing significantly inhibited CRC cell migration compared to the negative control (NC) siRNA in a wound-healing assay (Fig. 2a). Similarly, CRC cells with TUG1 knockdown exhibited lower migration into the lower portion of the transwell chamber than control cells (Fig. 2b). These data suggest that TUG1 may have a critical role in CRC migration and invasion, characteristic behaviors associated with metastasis.

### TUG1 is critical for TGF-β-induced migration and invasion in CRC cell lines

**Fig. 3:**
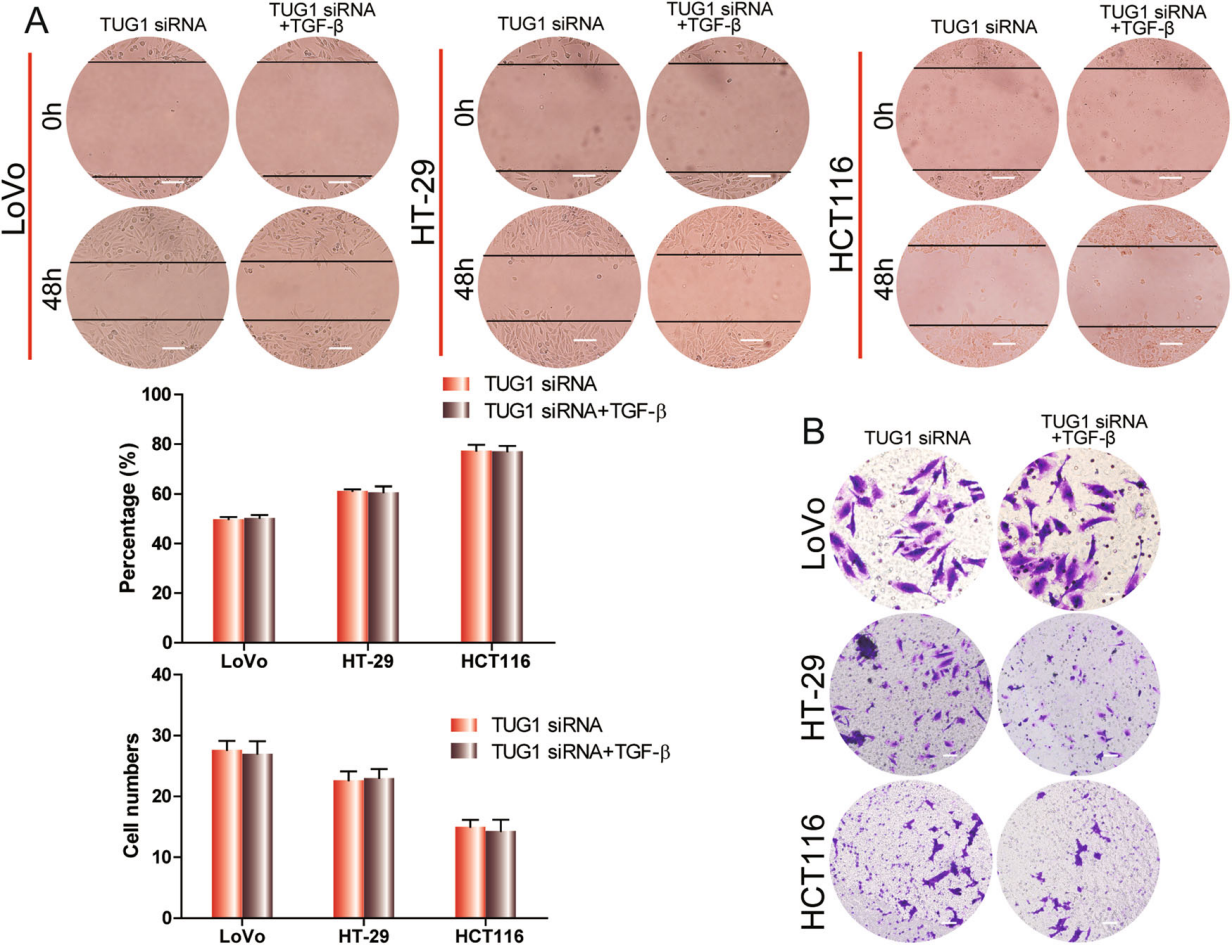
TUG1 mediates TGF-β-induced migration and invasion in CRC cell lines. **a**, **b** Wound-healing assay (scale bar, 50 μm) and transwell invasion analysis (scale bar, 100 μm) of CRC cells transfected with TUG1 siRNA and treated with 3 ng/ml TGF-β for 48 h.

To determine whether TUG1 has an indispensable role in TGF-β pathway to regulate invasion and migration in CRC cells, we treated TUG1-knockout CRC cells with TGF-β and evaluated their invasion and migration. TGF-β treatment did not change wound-healing capacity (migration) or invasion through matrix-coated transwells of CRC cells with TUG1 knockdown (Fig. 3a, b).

### TGF-β regulates EMT by targeting TUG1 in CRC cells

**Fig. 4:**
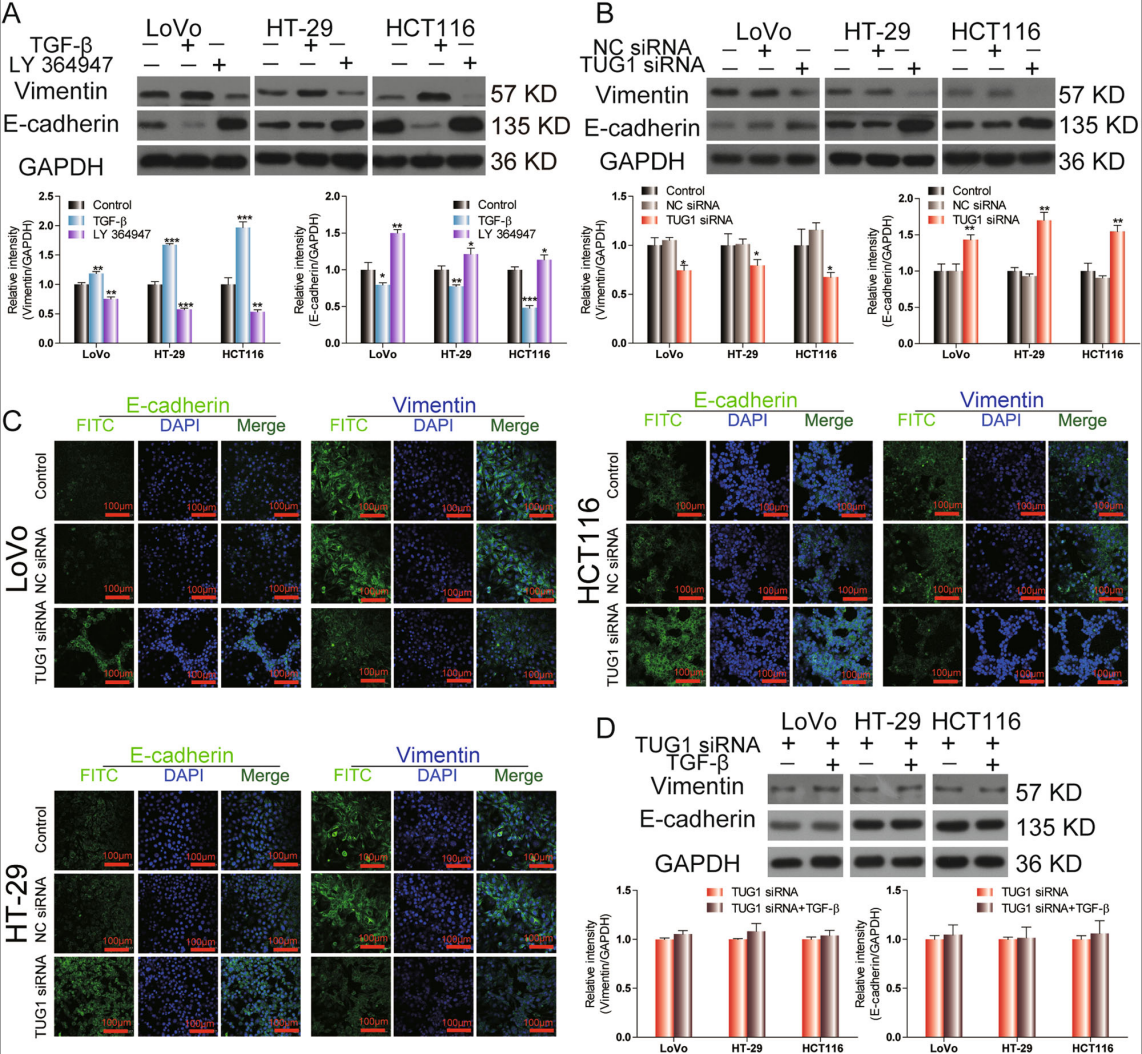
TUG1 regulates EMT in CRC cells. **a** The expression levels of vimentin and E-cadherin in LoVo, HT-29, and HCT116 cells were detected by western blot following treatment with 3 ng/ml TGF-β or 2 μm LY 364947 for 48 h. *P < 0.05, **P < 0.01, and ***P < 0.001, versus control. **b**, **c** Expression of vimentin and E-cadherin in LoVo, HT-29, and HCT116 cells transfected with NC-siRNA or TUG1 siRNA was analyzed by western blot and immunofluorescence, *P < 0.05 and **P < 0.01, versus NC-siRNA. Scale bar, 100 μm. **d** LoVo, HT-29, and HCT116 cells transfected with TUG1 siRNA were treated with 3 ng/ml TGF-β and expression of vimentin and E-cadherin was evaluated by western blot.

During the EMT process, the epithelial marker E-cadherin is downregulated, and mesenchymal markers are elevated^16^. In LoVo, HT-29, and HCT116 cells, the protein expression levels of the mesenchymal marker vimentin were significantly upregulated after TGF-β treatment, and E-cadherin expression was downregulated (Fig. 4a). Treatment with LY 364947 reduced vimentin expression and enhanced E-cadherin expression (Fig. 4a). Compared with the NC-siRNA group, TUG1 silencing significantly reduced vimentin expression and enhanced E-cadherin expression (Fig. 4b). Treatment with TGF-β after TUG1 knockdown did not increase vimentin expression and did not decrease E-cadherin expression (Fig. 4d). Consistently, TUG1 siRNA increased expression of E-cadherin and decreased expression of vimentin, as assessed through immunofluorescence (Fig. 4c). These data suggest that TGF-β-induced EMT is mediated through TUG1 in CRC cells.

### TWIST1 is regulated by TUG1 in CRC cell lines

**Fig. 5:**
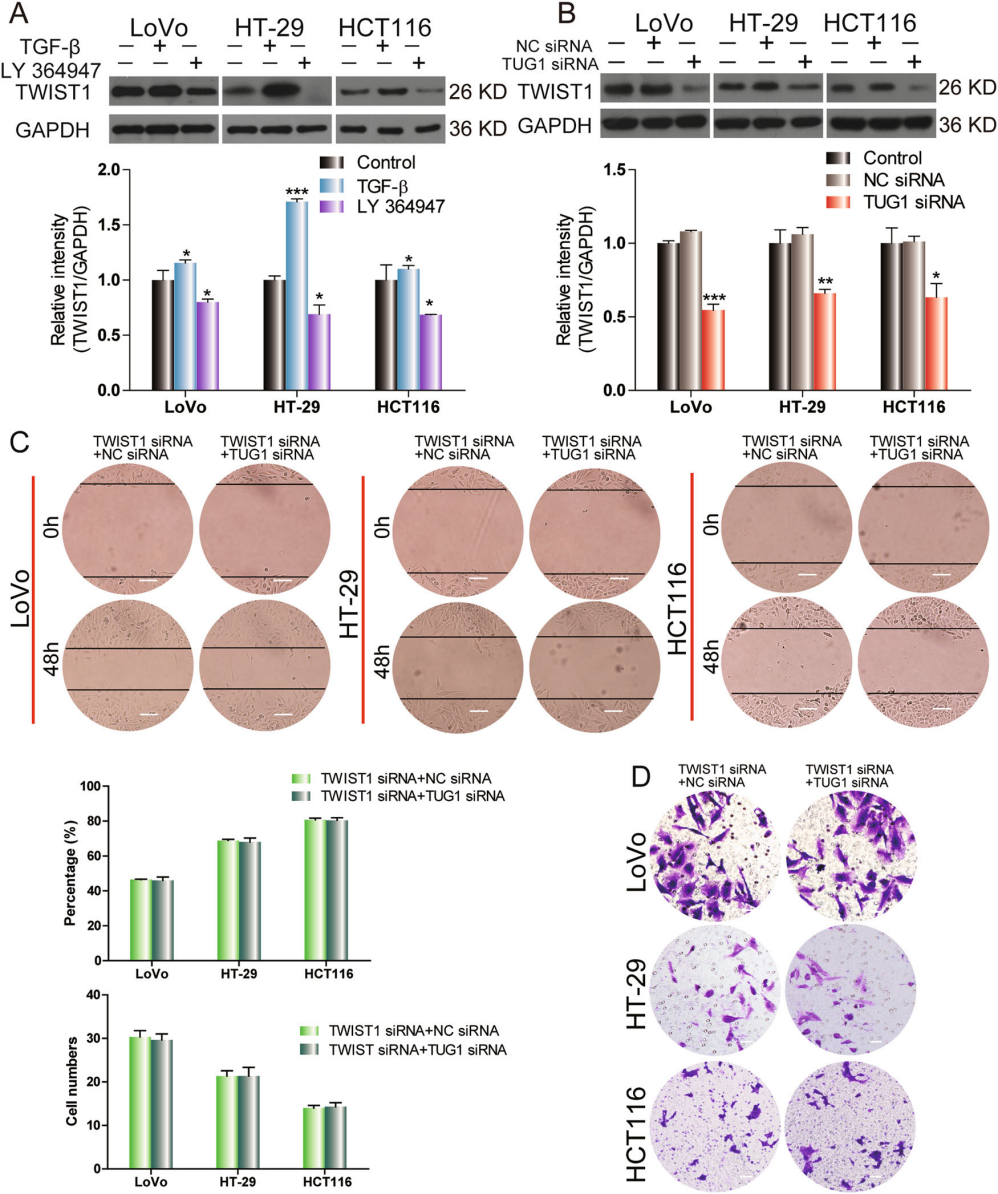
TUG1 regulates TWIST1 in CRC cell lines. **a** The expression levels of TWIST1 in LoVo, HT-29, and HCT116 cells were detected by western blot following treatment with 3 ng/ml TGF-β or 2 μm LY 364947 for 48 h, *P < 0.05 and ***P < 0.001, versus control. **b** TWIST1 expression in LoVo, HT-29, and HCT116 cells transfected with NC-siRNA or TUG1 siRNA was analyzed by western blot, *P < 0.05, **P < 0.01, and ***P < 0.001, versus NC-siRNA. **c**, **d** Wound-healing assay (scale bar, 50 μm) and transwell invasion analysis (scale bar, 100 μm) of CRC cells transfected with TUG1 siRNA were transfected with NC-siRNA or TWIST1-siRNA.

Western blot showed that the expression of TWIST1 was increased by TGF-β treatment and decreased by LY 364947 treatment in CRC cells (Fig. 5a). TUG1 siRNA significantly downregulated TWIST1 compared with NC-siRNA (Fig. 5b) in CRC cells. To study the role of TWIST1 in migration of CRC cells, siRNA was used to silence the expression of TWIST1. TWIST1 silencing significantly reduced migration of CRC cells, and inhibited EMT processes, as demonstrated by reduced vimentin expression and increased E-cadherin expression (Figs. S2 and S3). When both TWIST1 and TUG1 were simultaneously knocked down with siRNA, the wound healing and invasiveness of CRC cells remained constant (Fig. 5c, d). Collectively, these results suggest that TWIST1-mediated EMT is associated with TUG1 in CRC cells.

### TUG1 regulates CRC metastasis in vivo

**Fig. 6:**
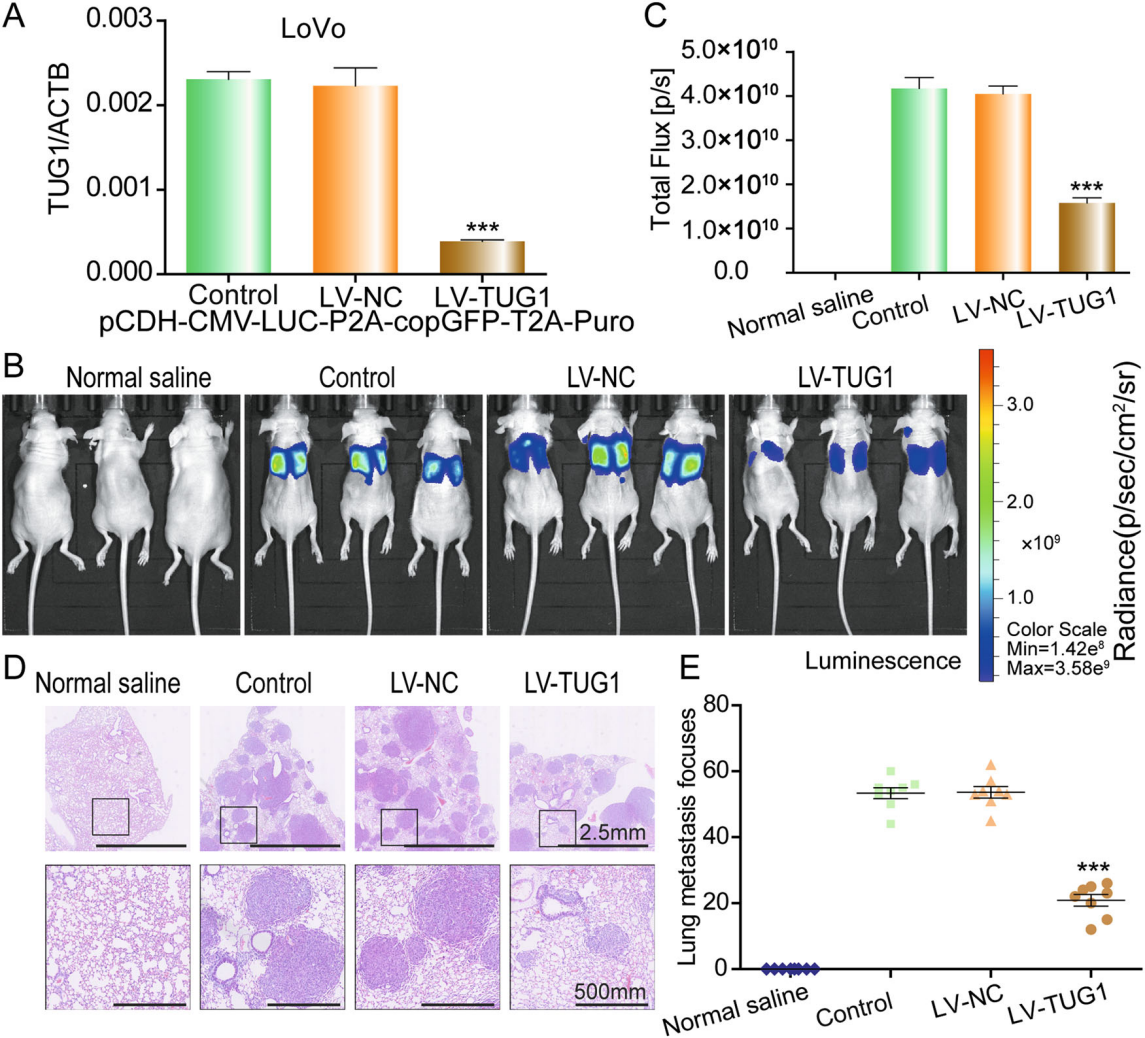
TUG1 promotes tumor metastasis in vivo. **a** QRT-PCR was used to confirm the effects of TUG1 knockdown after transfection of TUG1 shRNA in LoVo cells, compared with the LV-NC group: ***P < 0.001. **b** Representative images of luciferase expression from lung metastasis of the normal saline, control, NC, and shTUG1 groups are shown (*n* = 8 per group). **c** Quantification of the total flux, compared with the LV-NC group: ***P *<* 0.001. **d** HE staining of lungs from the normal saline, control, NC, and shTUG1 groups after injection of physiological saline or CRC cells transfected with NC-siRNA or TUG1 shRNA. **e** Quantification of lung metastatic foci, compared with the LV-NC group: ***P < 0.001.

To determine the influence of TUG1 expression levels on CRC metastasis in vivo, we employed a tail vein injection metastasis model. TUG1 shRNA was transfected into LoVo cells and transfection efficiency was determined by qRT-PCR (Fig. 6a). Control, NC, TUG1 shRNA luc-LoVo cells, and normal saline were injected into nude mice through the tail vein. Luciferase activity within the lungs of mice in the TUG1 shRNA group was significantly lower than the NC group 4 weeks after injection (Fig. 6b, c). Consistently, fewer metastatic foci were found in the lungs of mice in the TUG1 shRNA group compared with the NC group (Fig. 6d, e). These results suggest that knockdown of TUG1 suppresses metastasis of CRC cells in vivo.

## Discussion

TGF-β has a critical role in the regulation of many cellular processes, including proliferation, migration, invasion, and EMT^17^. However, TGF-β is not considered a good cancer target because it can act as either a tumor suppressor or a tumor promoter, depending on the context^18,19^. Therefore, it is important to develop novel therapies to regulate the downstream signaling components of TGF-β that are cancer promoting. Recently, large quantities of lncRNAs, including maternally expressed gene 3 (MEG3), BRAF-activated noncoding RNA (BANCR), and metastasis-associated Lung adenocarcinoma transcript 1 (MALAT1) were reported to regulate TGF-β signaling^20^. In the present study, we demonstrated that TGF-β treatment promotes migration and significantly upregulates TUG1 in CRC cell lines, whereas LY 364947 reduced migration and suppressed TUG1.

TUG1 was originally discovered to play an important role in mouse retinal development, and was found to be dysregulated in many cancers, including non-small cell lung cancer, CRC, hepatocellular carcinoma (HCC), and gastric cancer (GC)^21^. Our data demonstrate that TUG1 silencing significantly inhibits migration and invasion in three CRC cell lines, which is consistent with previous reports^15,22^. Moreover, the addition of TGF-β could not reverse TUG1-knockout-induced inhibition of migration and invasion, suggesting that TUG1 is a downstream component of TGF-β signaling, and might be a potential therapeutic target against CRC metastasis. Furthermore, we found that TUG1 is upregulated in CRC cancer tissue compared with adjacent normal tissue. These results demonstrate that TUG1 may be a regulator of CRC pathogenesis.

TGF-β can stimulate EMT by decreasing the expression of epithelial markers, such as E-cadherin, and increasing the expression of mesenchymal markers, such as N-cadherin, fibronectin, and vimentin^19,23^. Therefore, we further investigated the expression of vimentin and E-cadherin in CRC cells by western blot and immunofluorescence. Consistently, our results showed that TGF-β treatment upregulated vimentin expression and downregulated E-cadherin expression, whereas LY 364947 resulted in an opposite effect. Furthermore, TUG1 silencing increased E-cadherin expression and reduced vimentin expression, and the addition of TGF-β could not reverse these effects. These data suggest that TUG1-knockout can block TGF-β-mediated EMT in CRC cells.

TWIST1 is an important transcription factor that belongs to the basic-helix-loop-helix (bHLH) family and induces EMT in CRC cells^24,25^. Notably, we found that TWIST1 silencing significantly inhibited migration and EMT processes. In addition, TGF-β promoted TWIST1 expression, whereas TUG1 silencing significantly inhibited TWIST1 expression. Furthermore, simultaneous knockdown of TUG1 and TWIST1 in CRC cells showed no synergistic or additive impact on migration and invasion. These results indicated that TWIST1 is a downstream target of TUG1 in CRC, and suggest that TUG1 is a key component of the TGF-β/TWIST1/EMT signaling axis.

As TUG1 knockdown played an anti-metastasis role in vitro by inhibiting the TGF-β/TWIST1/EMT signaling pathway, we further investigated the impact of TUG1 knockdown in vivo. We found that TUG1 silencing significantly inhibited lung metastasis of CRC cells. Depending on the stage of primary CRC, lung metastasis can occur in 10–20% of patients^26^. Although significant progress has been made in the treatment of metastatic CRC, surgical resection for pulmonary metastasis, when possible, is still considered to be the optimal treatment^27,28^. Our results provided evidence that TUG1 may be a potential target to inhibit CRC lung metastasis.

Here, we provide the first report TUG1 is a downstream component of TGF-β signaling. We report that knockdown of TUG1 suppressed metastasis in CRC cells, both in vitro and in vivo. Furthermore, we demonstrate that TGF-β promotes metastasis of CRC via a TUG1/TWIST1/EMT signaling pathway. We suggest that TUG1 is be a potential target downstream of the TGF-β pathway that could be exploited in the treatment of CRC.

## Materials and methods

### Drugs and reagents

TGF-β (TGF-β-1, cat. no. 100-21) was purchased from PeproTech and LY 364947 (cat. no. S2805) was purchased from Selleck Chemicals. The antibodies used for western blot and immunofluorescence staining were as following: anti-TWIST1 antibody (cat. no. 46702), anti-vimentin antibody (cat. no. 5741), anti-E-cadherin antibody (cat. no. 3195), anti-GAPDH antibody (cat. no. 2118), and horseradish peroxidase (HRP)-linked secondary antibody goat anti-rabbit lgG (cat. no. 7074); all antibodies were obtained from Cell Signaling Technology.

### Patients and sample collection

Fresh CRC tissues and paired adjacent tissues were obtained from 27 patients undergoing surgical procedures at Affiliated Hospital of Jiaxing College, between December 2017 and March 2018. All samples were stored at − 80 °C in order to avoid degradation of RNA. Before the use of these clinical materials for research, written consents from all patients and approval of Affiliated Hospital of Jiaxing College Ethic Review Committees were obtained.

### Cell culture

CRC cell lines (LoVo, cat. no. TCHu 82; HT-29, cat. no. TCHu103; HCT116, cat. no. TCHu 99) were purchased from the Shanghai Institute of Biochemistry and Cell Biology (Shanghai, China). LoVo and HT-29 cells were maintained in RPMI-1640 medium supplemented with 10% fetal bovine serum (FBS), and HCT116 cells were cultured in McCoy’s 5 A medium (modified) containing 10% FBS. All cells were cultured in a humidified atmosphere at 37 °C with 95% air and 5% CO_2_. The source of cell lines was recently authenticated by short tandem repeats profiling and tested for mycoplasma contamination.

### Western blot

Cells were harvested in lysis buffer (Cell Signaling Technology, Beverley, MA) supplemented with a protease inhibitor and phosphatase inhibitor (Sigma, St. Louis, MO). Cell lysates were centrifuged at 12,000 r.p.m. for 30 min at 4 °C and the supernatants were carefully collected. Total protein concentration was determined by bicinchoninic acid assay (Beyotime Biotech, Haimen, China). Equal amounts of total protein from each sample were separated by sodium dodecyl sulfate polyacrylamide gel electrophoresis and transferred to polyvinylidene difluoride membranes (Millipore, Billerica, MA). The membranes were blocked with 5% milk in Tris-buffered saline supplemented with 0.1% Tween-20 for 1 h, and were then incubated with primary antibodies at 4 °C overnight. After incubation with HRP-conjugated secondary antibodies, protein bands were detected using an enhanced chemiluminescence kit (GE Healthcare Life Sciences, Little Chalfont, UK) and visualized on autoradiography film.

### RNA extraction and qRT-PCR

Total RNA from CRC cells was extracted using TRIzol reagent (Invitrogen), according to the manufacturer’s instructions. Total RNA was then reverse transcribed to cDNA using the TaqMan Reverse Transcription Kit (Applied Biosystems). Gene expression analysis was performed by qRT-PCR using a SYBR Premix Ex Taq Kit (Takara, Dalian, China). Relative gene expression was quantified using the comparative threshold cycle (2^−ΔΔCt^) method. The sequences of the primers used in qRT-PCR (Shanghai Sangon Biological Engineering Technology Services Co., Ltd) are as follows:

TUG1,

TUG1-F 5′-GCCCGAGATGATTCCTACCA-3′

TUG1-R 5′-ACAGGAGTGGAGGTAAAGGC-3′

TWIST1,

TWIST1-F 5′-GCAAGAAGTCGAGCGAAGAT-3′

TWIST1-R 5′-GCTCTGCAGCTCCTCGAA-3′

ACTB,

ACTB-F 5ʹ-TGGCACCCAGCACAATGAA-3ʹ,

ACTB-R 5ʹ-CTAAGTCATAGTCCGCCTAGAAGCA-3ʹ.

### Small interfering RNA (siRNA) transfection

Cells (1 × 10^5^ per well) were seeded in six-well plates. The following day, cells were washed two times with OPTIM-MEM (Gibco, Massachusetts, USA) and transfected with siRNA (final concentration 100 nm) using Lipofectamine 2000 (Invitrogen, Carlsbad, CA), according to the manufacturer’s instructions. FBS was added to cells to a final concentration of 10% after 6 h. The TUG1 siRNA TUG1-homo-1361 proved to effectively knockdown TUG1 in vitro, and was packaged into lentivirus (Hanbio, Shanghai, China) for in vivo studies. Transfectants were screened by puromycin selection to select for cells that were stably transfected with TUG1-homo-1361 siRNA. Selected cells were collected and cultured for in vivo experiments. The sequences of the TUG1 siRNAs and TWIST1 siRNAs are as follows:

TUG1-homo-557, 5′-GGUUGGUUGUGGGAUUUCUTT-3′

5′-AGAAAUCCCACAACCAACCTT-3′

TUG1-homo-1361, 5′-CCCGUCAACUCUGUUAUCUTT-3′

5′-AGAUAACAGAGUUGACGGGTT-3′

TUG1-homo-2547, 5′-CUCCAUCCAAAGUGAAUUATT-3′

5′-UAAUUCACUUUGGAUGGAGTT-3′

TWIST1-homo-1575, 5′-GGUGUCUAAAUGCAUUCAUTT-3′

5′-AUGAAUGCAUUUAGACACCTT-3′

TWIST1-homo-810, 5′-GGUACAUCGACUUCCUCUATT-3′

5′-UAGAGGAAGUCGAUGUACCTT-3′

TWIST1-homo-780, 5′-GCAAGAUUCAGACCCUCAATT-3′

5′-UUGAGGGUCUGAAUCUUGCTT-3′

Negative control (NC), 5′-UUCUCCGAACGUGUCACGUTT-3′

5′-ACGUGACACGUUCGGAGAATT-3′.

### Immunofluorescence staining

CRC cells were washed three times with PBS, fixed with 4% paraformaldehyde for 15 min, washed three times with PBS, and blocked with 5% BSA for 30 min. Anti-E-cadherin or anti-vimentin antibody was diluted 1:200 in 0.5% BSA and wells were incubated in primary antibody overnight at 4 °C. After incubation with secondary antibodies diluted 1:200 in 0.5% BSA for 2 h at room temperature, cells were incubated with 0.1% 4′,6-diamidino-2-phenylindole for 5 min. Cells were washed three times with PBS, and were then examined using a confocal microscope (ZEISS LSM800).

### Animal studies

Six-week-old BALB/c-nu male nude mice (GemPharmatech Co., Ltd, Nanjing, China) were randomly divided into normal saline, control, NC (noncoding siRNA), and shRNA-TUG1 groups (eight mice per group). In all, 2.0 × 10^6^ LoVo cells were suspended in physiological saline solution and injected via tail vein. Four weeks later, mice were injected intraperitoneally with d-luciferin (75 mg/kg), and photographed within 30 min. Mice were killed and lungs were isolated for hematoxylin-eosin (HE) staining. All animal studies were approved by the Animal Care Ethics Committee of first Affiliated Hospital, Zhejiang University and performed in accordance with the institutional guidelines.

### HE staining

Lungs collected from the nude mice were fixed with 4% paraformaldehyde for 24 h. After dehydration and paraffin embedding, the samples were sectioned at 4 µm thickness and stained with hematoxylin solution for 4 min. Then the sections were stained with eosin solution for 2 min and dehydrated with graded ethanol. The slides were mounted and photographed using a Nanozoomer 2.0-RS fluorescence microscope (Hamamatsu, Japan). The number of metastatic foci in the lungs was counted.

### Wound-healing assay

Cells (3 × 10^5^ per well) were seeded in six-well plate and grown to confluent monolayers. Wounds were created using a 200 μl sterile pipette tip. Cellular debris and floating cells were removed, and serum-free medium containing the indicated concentrations of TGF-β or LY 364947 was added to the wells. Images were captured 0 h and 48 h after wounding, and the wound area was quantified to estimate wound closure.

### Transwell invasion analysis

CRC cells (5 × 10^4^ per well) in 200 μl serum-free medium were seeded in the upper chambers (24-well insert, 8 mm, Corning, NY) of transwell inserts that were coated with Matrigel (BD Biosciences, San Jose, CA). As chemoattractant, 700 μl culture medium supplemented with 10% FBS was added into the lower chambers. After incubation at 37 °C for 24 h, the cells that migrated through the pores in the transwell membrane were fixed with 4% paraformaldehyde for 30 min, stained with 0.1% crystal violet for 10 min, and counted.

### Statistical analysis

All data are presented as means ± SD from three independent experiments and were analyzed using GraphPad Prism 5 (GraphPad, San Diego, CA). Differences between groups were analyzed by Student’s *t* test and considered statistically significant when *P* < 0.05.

## Supplementary information

Supplement

Supplement Figure 1

Supplement Figure 2

Supplement Figure 3
