# Supplementary material for: The long noncoding RNA TUG1 is required for TGF-β/TWIST1/EMT-mediated metastasis in colorectal cancer cells
Source: Cell Death Dis. 2020 Jan 27;11(1):65. doi: 10.1038/s41419-020-2254-1 (PMC6985237; doi:10.1038/s41419-020-2254-1)
Supplement: Supplementary file 1 — Supplement [file 41419_2020_2254_MOESM1_ESM.docx]

**The long non-coding RNA TUG1 is required for TGF-β/TWIST1/EMT-mediated metastasis in colorectal cancer cells**

**Supplementary materials and methods**

**Cell viability assay**

Cell viability was measured by Cell Counting Kit 8 (CCK-8; Dojindo, Kumamoto, Japan) assay. LoVo, HT-29 and HCT116 cells were seeded into 96-well plates and treated with 3 ng/ml TGF-β or 2 μM LY 364947 for 48 h. CCK-8 solution (10 μl per well) was added to cells and incubated for 2 h at 37°C. The absorbance was measured with an MRX II microplate reader (Dynex, Chantilly, VA, USA) at 450 nm.

**Supplementary figure legends**

**Fig.S1** **Viability of CRC cell lines subsequent to TGF-β and LY 364947 treatment.** (a) CCK-8 assay was used to detect the cell viability in LoVo, HT-29 and HCT116 cells after treatment of 3 ng/ml TGF-β or 2 μM LY 364947 for 48 h.

**Fig.S2 TWIST1 mediate metastasis in CRC cell lines.** (a) QRT-PCR was used to confirm the effects of TWIST1 knockdown after transfection of TWIST1 siRNA in LoVo, HT-29 and HCT116 cells, compared with the NC siRNA group: ***P* < 0.01 and ****P* < 0.001. (b) Wound healing assay of LoVo, HT-29 and HCT116 cells transfected with NC-siRNA or TWIST1-siRNA. The ratio between the residual gap at 48 h and the initial gap at 0 h was calculated, ***P* < 0.01 and ****P* < 0.001, versus NC siRNA. Scale bar, 50 μm.

**Fig.S3 EMT was mediated by TWIST1 in CRC cell lines.** (a) Expression of vimentin and E-cadherin in LoVo, HT-29 and HCT116 cells transfected with NC-siRNA or TWIST1-siRNA was analyzed by immunofluorescence analysis. Scale bar, 100 μm. (b) The expression levels of TWIST1, vimentin and E-cadherin in LoVo, HT-29, and HCT116 cells were detected by western blot, **P* < 0.05, ***P* < 0.01 and ****P* < 0.001, versus NC siRNA.
