# Supplementary figures and images for: The long noncoding RNA TUG1 is required for TGF-β/TWIST1/EMT-mediated metastasis in colorectal cancer cells
Source: Cell Death Dis. 2020 Jan 27;11(1):65. doi: 10.1038/s41419-020-2254-1 (PMC6985237; doi:10.1038/s41419-020-2254-1)

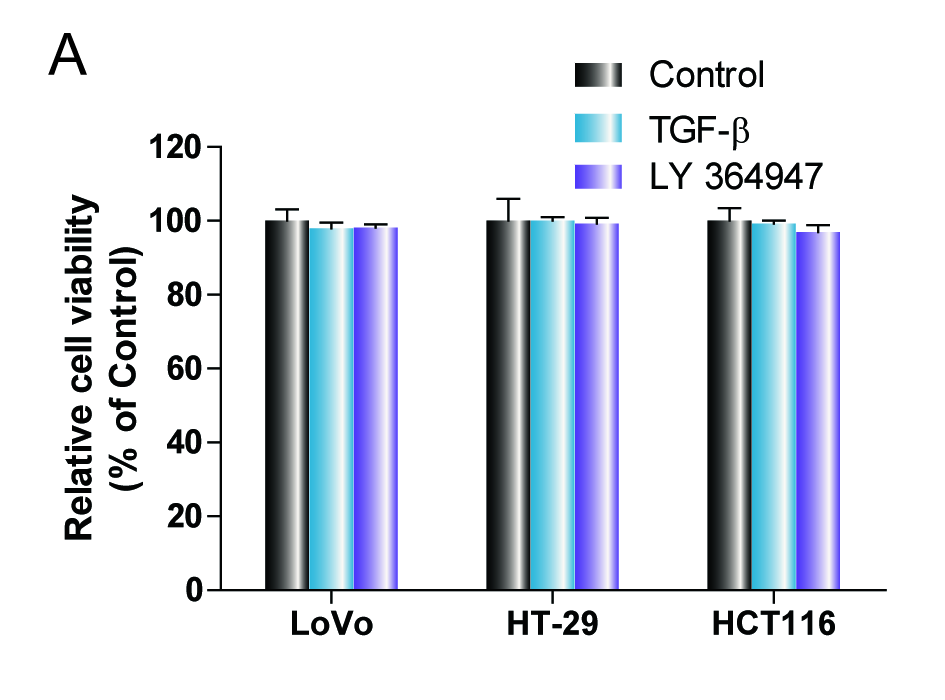

Supplement: Supplementary file 2 — Supplement Figure 1 [file 41419_2020_2254_MOESM2_ESM.tif]

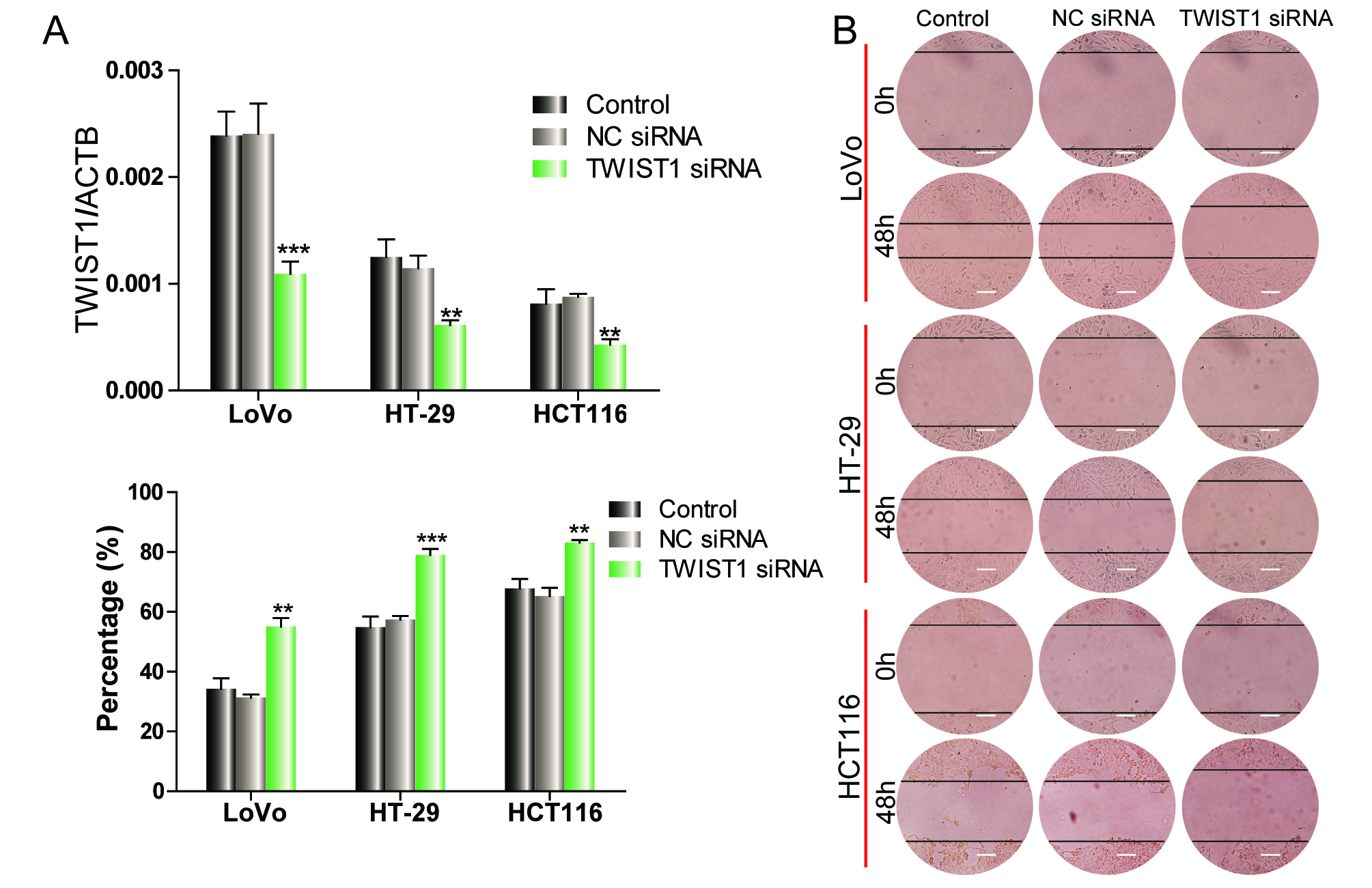

Supplement: Supplementary file 3 — Supplement Figure 2 [file 41419_2020_2254_MOESM3_ESM.tif]

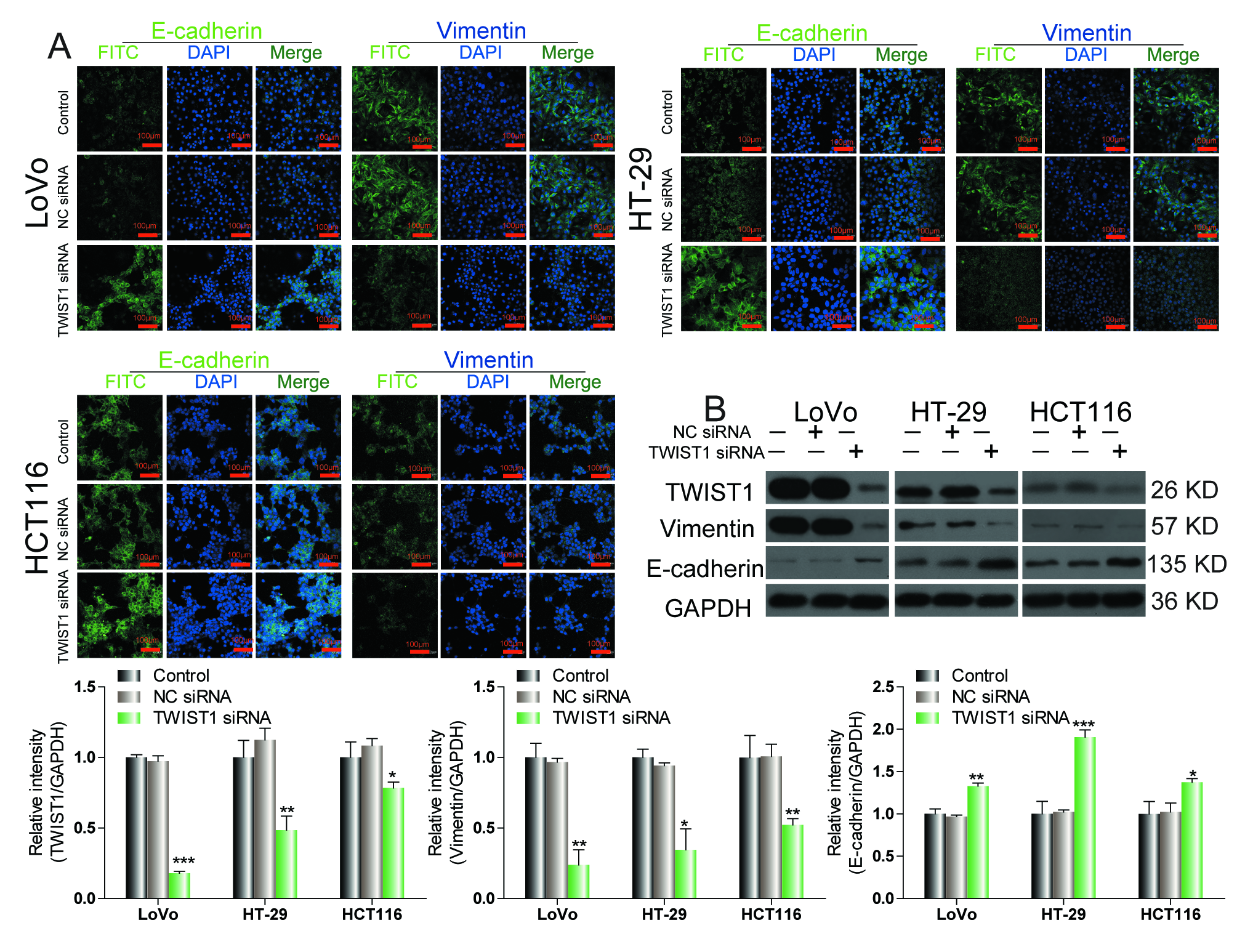

Supplement: Supplementary file 4 — Supplement Figure 3 [file 41419_2020_2254_MOESM4_ESM.tif]
